# Functional testing, coronary artery calcifications, and outcomes in Hodgkin lymphoma survivors treated with chest radiation

**DOI:** 10.1186/s40959-023-00157-2

**Published:** 2023-01-20

**Authors:** Sanjay Divakaran, Diana M. Lopez, Sean M. Parks, Jon Hainer, Andrea K. Ng, Ron Blankstein, Marcelo F. Di Carli, Anju Nohria

**Affiliations:** 1 Cardiovascular Imaging Program, Departments of Medicine and Radiology, Brigham and Women’s Hospital, Boston, MA USA; 2 Division of Cardiovascular Medicine, Brigham and Women’s Hospital, Brigham & Women’s Hospital, 75 Francis Street, Boston, MA 02115 USA; 3 Harvard Medical School, Boston, MA USA; 4 Department of Radiation Oncology, Brigham and Women’s Hospital, Boston, MA USA

**Keywords:** Cancer survivorship, Cardio-oncology, Coronary artery calcifications, Hodgkin lymphoma, Primary prevention, Stress testing

## Abstract

**Background:**

Consensus guidelines recommend periodic screening for coronary artery disease (CAD) in Hodgkin lymphoma (HL) survivors treated with radiation therapy (RT) to the chest. However, the prognostic utility of screening strategies in this population remains unclear. We evaluated the association between functional testing, coronary artery calcifications (CAC), and guideline-based risk assessment and major adverse cardiovascular events (MACE) in HL survivors treated with RT.

**Methods:**

We retrospectively studied HL survivors treated with RT who underwent functional testing between 2003 and 2020 and chest computed tomography (CT) within 12 months of each other at our center. CAC was assessed semi-quantitatively from CT images. Cardiovascular risk was estimated using the 2019 ACC/AHA Guideline on the Primary Prevention of Cardiovascular Disease. Diagnostic test characteristics were calculated using major adverse cardiac events (MACE) during follow-up as the gold standard.

**Results:**

The study included 159 patients (median age at functional testing 48 years, median age at HL diagnosis 27 years, 62.9% female). Abnormal functional testing had the highest specificity (94.2% (95% CI 88.4%-97.6%)) and positive likelihood ratio (4.55 (95% CI 1.86–11.13)) while CAC had the highest sensitivity (63.2% (95% CI 46.0%-78.2%)) and lowest negative likelihood ratio (0.52 (95% CI 0.34–0.80)). Specificity for ACC/AHA risk assessment was also high (88.5% (95% CI 81.1%-93.7%)). Over 3.3 years of follow-up, abnormal functional testing (adjusted subdistribution hazard ratio (SHR) 5.10, 95% CI 2.41 – 10.78, *p* < 0.001) and CAC (adjusted SHR 3.58, 95% CI 1.35 – 9.47, *p* = 0.010) were both significantly associated with MACE.

**Conclusions:**

In HL survivors treated with RT, both abnormal functional testing and ACC/AHA risk assessment had high specificity for subsequent MACE, but CAC had higher sensitivity. Further research is needed to inform CAD screening and primary prevention strategies in this population.

**Supplementary Information:**

The online version contains supplementary material available at 10.1186/s40959-023-00157-2.

## Background

Most patients with Hodgkin lymphoma (HL) are diagnosed between the ages of 15 and 30 years. Due to tremendous progress in the treatment and management of HL, 80% of patients now have curable disease [1]. However, survivors need to be actively followed for adverse late-effects of cancer treatment. For example, HL survivors treated with radiation therapy to the chest are at risk for the development of secondary thoracic malignancies and coronary artery disease (CAD) [2, 3].

Because radiation-associated CAD is usually observed more than 5–10 years post-treatment, the National Comprehensive Cancer Network Clinical Practice Guidelines recommend a baseline stress test and echocardiogram 10 years after treatment for HL survivors treated with chest radiation [1]. A consensus statement from the European Association of Cardiovascular Imaging and the American Society of Echocardiography recommends functional stress testing 5–10 years post-treatment, and reassessment every five years subsequently for asymptomatic patients with any malignancy treated with chest radiotherapy, who are considered high-risk [4]. Additionally, because HL survivors treated with radiation therapy have been shown to have worse long-term outcomes after a cardiovascular event when compared with matched patients, [5] strategies for the primary prevention of cardiovascular events before they occur are of high importance. However, consensus primary prevention guidelines do not directly address statin therapy in this population [6].

In this study, we aimed to better understand the prognostic utility of functional imaging, incidental coronary artery calcifications (CAC) on chest computed tomography (CT) imaging, and cardiovascular risk assessment based on the 2019 American College of Cardiology (ACC)/American Heart Association (AHA) Guideline on the Primary Prevention of Cardiovascular Disease [6] in survivors of HL treated with chest radiation therapy and without a known history of CAD.

## Methods

### Study population

The study population included consecutive patients with a history of HL, treated with chest radiation, who underwent functional testing for CAD between 2003 and 2020 at Brigham and Women’s Hospital in Boston, Massachusetts and had available images from a CT chest within 12 months of functional testing. The cohort was initially identified using International Classification of Diseases (ICD) -9 and ICD-10 codes to identify patients with a history of HL. After detailed review of each patient’s longitudinal electronic health record (blinded to imaging results and outcomes) to confirm a diagnosis of HL and the absence of CAD, patients without a history of HL, patients with a history of clinically overt CAD (defined as a history of myocardial infarction (MI), percutaneous coronary intervention (PCI), or coronary artery bypass graft (CABG) surgery), and patients with prior invasive coronary angiography were excluded. Patients referred for functional testing for non-CAD screening (such as a dobutamine stress echocardiography for further assessment of aortic stenosis), incomplete functional testing, functional testing performed before HL diagnosis, prior orthotopic heart transplantation, or with non-accessible CT chest images were excluded. After excluding patients who did not receive chest radiation therapy, the final cohort consisted of 159 patients (Supplemental Fig. 1).

**Fig. 1:**
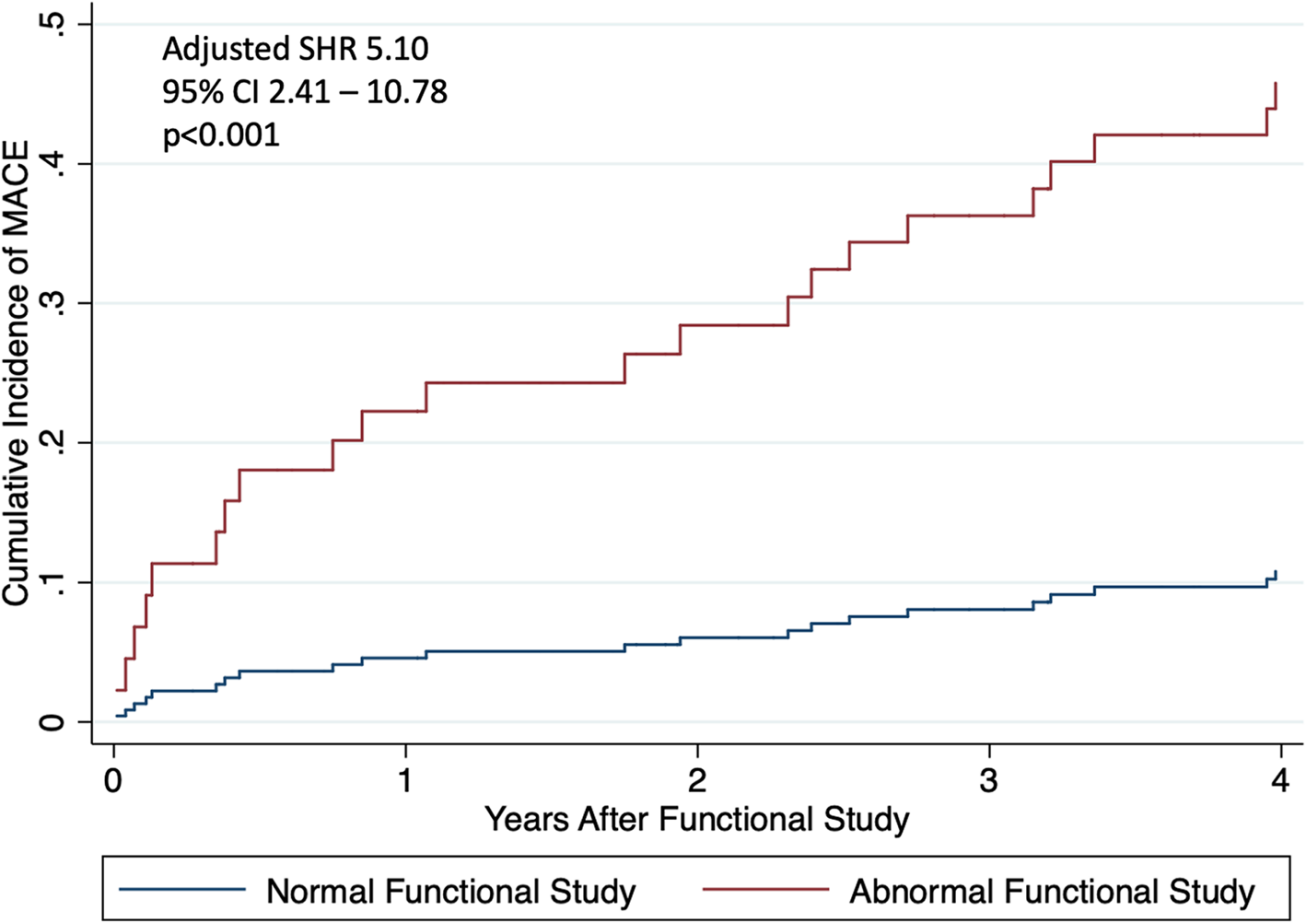
Time to Incident Major Adverse Cardiovascular Event by Functional Testing Result. Cumulative incidence of major adverse cardiovascular events for the cohort is presented stratified by functional testing result. Multivariable analysis (considering competing risk of death) adjusted for the presence of coronary artery calcification, years between Hodgkin lymphoma diagnosis and functional testing, and Morise score. CI = confidence interval. MACE = major adverse cardiovascular event. SHR = subdistribution hazard ratio

Patient demographics and indications for functional testing and CT imaging were collected prospectively at the time of testing. Blood pressure, body mass index (BMI), medications, and risk factors were obtained prospectively at the time of functional testing. Lipid profiles were included if available within 12 months of functional testing.

### Functional testing

We categorized each functional test result as abnormal (positive or inconclusive) or normal (negative) using conventional criteria [7, 8]. Abnormal functional testing included positive or inconclusive (using conventional criteria [8, 9]) exercise treadmill test results, stress echocardiograms with a resting or stress-induced wall motion abnormality, and abnormal myocardial perfusion imaging (summed stress score > 2). If a patient had more than one functional study during the study period, the earliest functional study with an available paired CT within 12 months was included.

### Coronary artery calcification assessment

The presence and severity of CAC was assessed using a previously published semi-quantitative visual analysis of CT imaging [10, 11]. In most cases, these were non-electrocardiogram-gated CT scans. However, if formal CAC scoring was performed at the time of functional imaging, these data were incorporated (Agatston score 0 = none; 1–100 = mild CAC, 101–399 = moderate CAC, ≥ 400 = severe CAC).

### Guideline-directed cardiovascular risk assessment

Cardiovascular risk and recommendations regarding primary prevention statin therapy were retrospectively assessed at the time of functional testing using the 2019 ACC/AHA Guideline on the Primary Prevention of Cardiovascular Disease [6]. If the guidelines would have recommended a risk discussion with the patient regarding at least moderate-intensity statin therapy, the patient was classified as the guidelines recommending statin therapy. If the decision tree for a specific patient required a lipid profile for risk assessment and there was not one available within 12 months of functional testing, a guideline-directed recommendation regarding statin therapy was not given for that patient for this analysis.

Additionally, for patients whose age at HL diagnosis was less than 21-years-old and whose age at the time of functional testing was less than 40-years-old, the Childhood Cancer Survivor Study (CCSS) Cardiovascular Risk Calculator was used to estimate the risk of ischemic heart disease (10-year risk and risk by the age of 50) [12].

### Outcomes and diagnostic evaluation of functional testing, CAC assessment, and cardiovascular risk assessment by primary prevention guidelines

Diagnostic test characteristics were calculated using major adverse cardiovascular events (MACE) during follow-up as the gold standard. Ascertainment of clinical endpoints was determined by blinded adjudication of the longitudinal medical record, Mass General Brigham Research Patient Data Registry, and the National Death Index. MACE was defined as a composite of cardiovascular death, coronary revascularization, or hospitalization for heart failure, nonfatal MI, or nonfatal ischemic stroke. Hospitalization for nonfatal MI or heart failure required a discharge note with a primary hospitalization diagnosis of MI and/or heart failure. In addition, only events meeting the 2018 Fourth Universal Definition of MI or defined clinical criteria for the presence of symptoms, signs, and escalation of therapy for heart failure, were classified as such [13]. In addition to the occurrence of MACE, patients were also evaluated for all-cause death following functional testing. Clinical endpoints were determined independently by two cardiologists who were blinded to imaging results.

The sensitivity, specificity, positive predictive value (the probability that a patient with a positive test actually has the disease), negative predictive value (the probability that a patient with a negative test actually does not have the disease), positive likelihood ratio (the probability of a true positive over false positive test) [14], and negative likelihood ratio (the probability of a false negative over true negative test) [14] along with exact (Clopper-Pearson) 95% confidence intervals (CI), were calculated for abnormal functional testing, the presence of CAC on CT imaging, and cardiovascular risk assessment based primary prevention guidelines in the entire cohort and in two subgroups: patients referred for testing due to symptoms (including perioperative testing) and patients who were asymptomatic and referred for functional testing due to consensus guideline-recommended CAD screening in HL survivors. Area under the curve with 95% asymptotic normal CIs and receiver operator characteristic (ROC) curves were calculated and constructed, respectively, for the entire cohort and both subgroups for the combined strategy (functional testing, CAC assessment, and guidelines-recommend statin therapy discussion) with the number of combined abnormal results (zero, one, two, or three) as the cut points.

### Statistical analysis

Categorical variables are reported as frequencies with percentages (%). Continuous variables are expressed as mean (± standard deviation) or median (interquartile range (IQR)). To study the effect of abnormal functional testing and the presence of CAC on incident MACE and account for competing risk of death in the full cohort and in the asymptomatic and symptomatic subsets, univariable Fine and Gray competing risks regression modeling was performed using available covariates [15]. To avoid overfitting the model, demographic and medical history variables were incorporated into the validated Morise clinical risk score, which includes age, sex, symptoms, estrogen status, diabetes, hypertension, smoking, hyperlipidemia, family history of CAD, and obesity, for estimating the pre-test probability of CAD [16]. Multivariable adjustment was performed using the Morise score, and any covariates not included in the Morise score that had significant univariable association with the outcome (excluding the 2019 ACC/AHA Guideline recommendations given overlap between the Morise score and the 10-year atherosclerotic cardiovascular disease (ASCVD) risk calculator). We constructed cumulative incidence curves by functional testing result and the presence or absence of CAC to illustrate time-to-MACE. Differences were tested with the Wald test [17]. Fine and Gray competing risk-adjusted subdistribution hazard functions, with multivariable adjustment using the previously identified covariates, were used to examine the association between MACE and functional testing results and the presence of CAC. Graphical methods and Schoenfeld residuals were used to verify that proportional hazards assumptions were met. All tests were 2-sided, and a *p*-value of < 0.05 was considered statistically significant. Statistical analysis was performed with the use of Stata version 14.2 (Statacorp, College Station, Texas).

## Results

### Characteristics of the study cohort and testing

Patient characteristics for the study cohort are detailed in Table 1. Among the 159 patients in the study cohort (median age at functional testing 48 years (IQR 42 – 56), median age at HL diagnosis 27 years (IQR 20 – 36), 62.9% female), the median radiation dose to the chest was 37.2 Gy (IQR 36 – 42) and 84 (57.1%) patients were treated with concomitant anthracycline chemotherapy. The mean Morise score for the cohort was 6.4 ± 2.6, and 52 (32.7%) patients had hypertension, 54 (34.0%) had dyslipidemia, and 4 (2.5%) had diabetes at the time of functional testing.

**Table 1:** Patient characteristics

	**Total Cohort *n* = 159**
**Age at HL diagnosis (years)**	27 (20 – 36)
**Female**	100 (62.9%)
**Race**	
White	151 (95.0%)
Black	4 (2.5%)
Other	4 (2.5%)
**Radiation dose to chest (Gray)**	37.2 (36 – 42)
Mantle radiation or cumulative dose ≥ 35 Gy	120 (88.9%)
**Chemotherapy**	108 (68.4%)
Anthracycline	84 (57.1%)
Anthracycline dose (mg/m^2^)	300 (200–300)
Cumulative anthracycline dose ≥ 250 mg/m^2^	40 (64.5%)
**Decade of treatment**	
1960–69	4 (2.5%)
1970–79	25 (15.7%)
1980–89	49 (30.8%)
1990–99	47 (29.6%)
2000–09	27 (17.0%)
2010–20	7 (4.4%)
**Age at time of functional study (years)**	48 (42 – 56)
**Years between HL diagnosis and functional study**	20 (11 – 28)
**Hypertension**	52 (32.7%)
**Dyslipidemia**	54 (34.0%)
**Diabetes**	4 (2.5%)
**Family history of premature CAD**	35 (22.0%)
**Former or current tobacco use**	27 (17.0%)
**BMI ≥ 30 kg/m**^**2**^	31 (19.5%)
**Morise score**	6.4 (2.6)
**Pre-test probability of CAD by Morise score**	
Low (0–8 points)	124 (78.0%)
Intermediate (9–15 points)	35 (22.0%)
High (16–24 points)	0 (0.0%)
**On aspirin therapy**	31 (19.5%)
**On statin therapy**	35 (22.0%)

Values are presented as median (interquartile range), mean (standard deviation) or n (%) as appropriate

*BMI* Body mass index, *CAD* Coronary artery disease, *HL* Hodgkin lymphoma

The most frequently ordered functional test for CAD evaluation was exercise stress echocardiography (*n* = 62 (39.0%)) and the most common indication for functional test referral was asymptomatic CAD screening (*n* = 77 (48.4%)). The most common symptom resulting in functional testing referral was dyspnea (*n* = 34 (21.4%)). CT chest with or without contrast were the most common CT chest studies (*n* = 98 (61.6%)) and the most common reason for CT chest referral was routine lymphoma follow-up imaging (*n* = 58 (36.5%)). Further test referral characteristics are listed in Table 2.

**Table 2:** Test characteristics

	**Total Cohort *n* = 159**
**Functional test type**	
Exercise stress echocardiography	62 (39.0%)
ETT-ECG	35 (22.0%)
Exercise stress SPECT	27 (17.0%)
Stress PET	19 (12.0%)
Pharmacologic stress SPECT	6 (3.8%)
Level I CPET	4 (2.5%)
Exercise converted to pharmacologic stress SPECT	3 (1.9%)
Stress cardiac MRI	2 (1.3%)
Dobutamine stress echocardiography	1 (0.6%)
**Functional test indication**	
Asymptomatic CAD screening	77 (48.4%)
Dyspnea	34 (21.4%)
Chest pain	26 (16.4%)
Pre-op	12 (7.6%)
Syncope	3 (1.9%)
Palpitations	2 (1.3%)
Other	5 (3.1%)
**CT chest type**	
CT Chest without Contrast	55 (34.6%)
CT Chest with Contrast	43 (27.0%)
FDG PET/CT	21 (13.2%)
Stress PET	19 (12.0%)
CT-PE	17 (10.7%)
CCTA	2 (1.3%)
Other	2 (1.3%)
**CT chest indication**	
Lymphoma follow up	58 (36.5%)
Dyspnea	26 (16.4%)
Transmission scan for stress PET	17 (10.7%)
Lung cancer screening	15 (9.4%)
Pulmonary nodule follow up	11 (6.9%)
Other cancer	9 (5.7%)
Mesothelioma	3 (1.9%)
Sarcoma	3 (1.9%)
Breast	1 (0.6%)
Esophageal	1 (0.6%)
Laryngeal	1 (0.6%)
Chest pain	6 (3.8%)
Pre-op	2 (1.3%)
Other	15 (9.4%)

Values are presented as n (%)

*CAD* Coronary artery disease, *CCTA* Coronary computed tomography angiography, *CPET* Cardiopulmonary exercise testing, *CT* Computed tomography, *ECG* Electrocardiogram, *ETT* Exercise tolerance test, *FDG* Fluorodeoxyglucose, *HL* Hodgkin lymphoma, *MRI* Magnetic resonance imaging, *PE* Pulmonary embolism, *PET* Positron emission tomography, *SPECT* Single-photon emission computed tomography

### Test results and outcomes

**Fig. 2:**
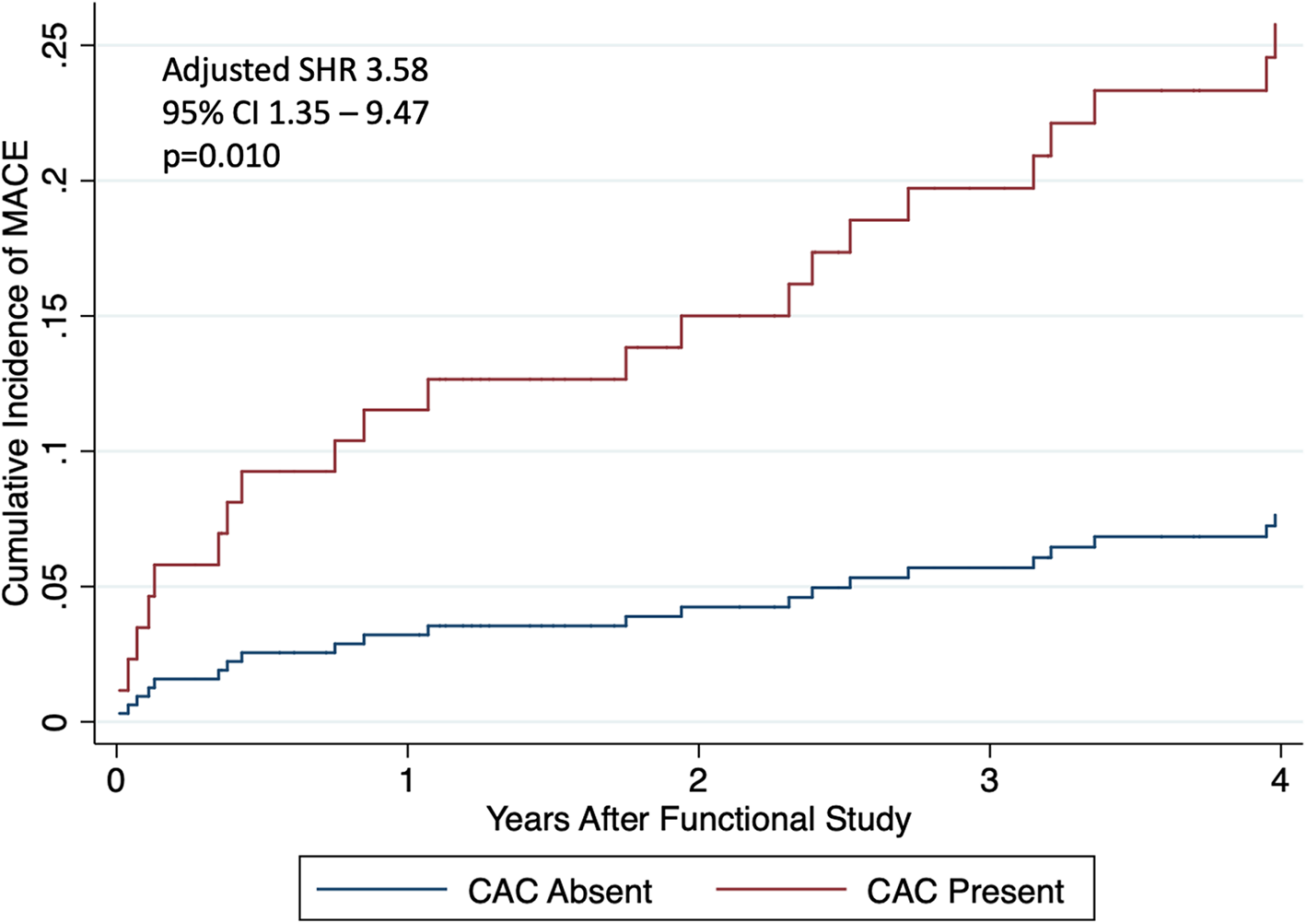
Time to Incident Major Adverse Cardiovascular Event by the Presence or Absence of Coronary Artery Calcifications. Cumulative incidence of major adverse cardiovascular events for the cohort is presented stratified by semi-quantitative coronary artery calcification assessment result. Multivariable analysis (considering competing risk of death) adjusted for abnormal functional testing, years between Hodgkin lymphoma diagnosis and functional testing, and Morise score. CAC = coronary artery calcifications. CI = confidence interval. MACE = major adverse cardiovascular event. SHR = subdistribution hazard ratio

**Fig. 3:**
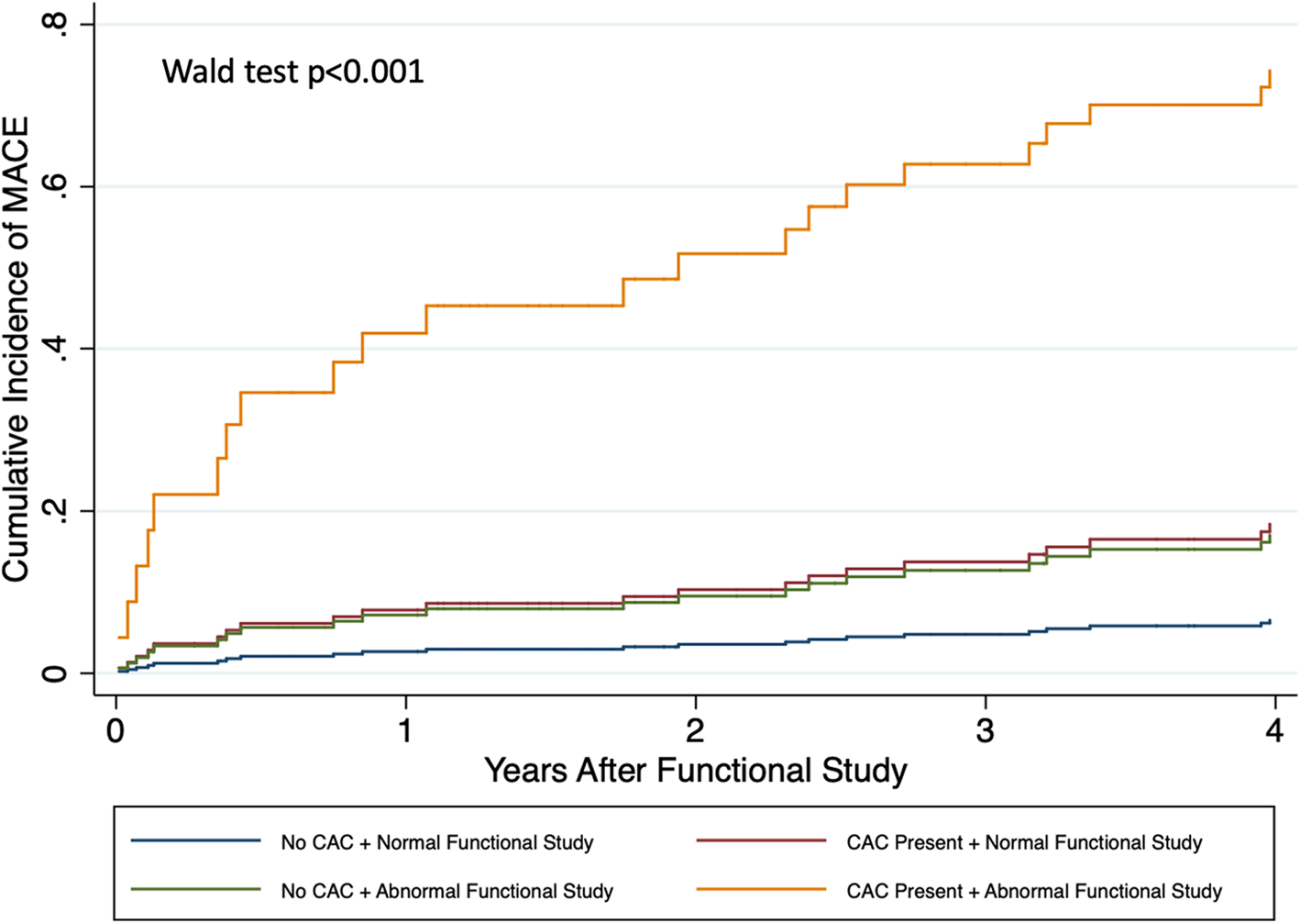
Time to Incident Major Adverse Cardiovascular Event by Functional Testing Result and Presence of Coronary Artery Calcifications. Cumulative incidence of major adverse cardiovascular events for the cohort is presented stratified by functional testing result and semi-quantitative coronary artery calcification assessment results. MACE = major adverse cardiovascular event

Among the 159 patients in the study cohort, 17 (10.7%) had abnormal functional testing and 59 (37.1%) had CAC present on CT chest (Table 3). Of the 142 patients who had a normal functional study, 50 (35.2%) had CAC present on CT chest (Supplemental Table 1). A total of 38 patients (23.9%) experienced a MACE over a median follow up of 3.3 years after functional testing (IQR 0.9 – 6.9) and 30.5 years after HL diagnosis (IQR 21 – 35) (18 coronary revascularizations, nine hospitalizations for heart failure, five hospitalizations for nonfatal MI, four hospitalizations for ischemic stroke, and two cardiovascular deaths) (Table 3). Both abnormal functional testing (Fig. 1) and the presence of CAC on CT imaging (Fig. 2) were significantly associated with incident MACE after multivariable adjustment (subdistribution hazard ratio (SHR) 5.10, 95% CI 2.41 – 10.78, *p* < 0.001 and SHR 3.58, 95% CI 1.35 – 9.47, *p* = 0.010, respectively) (Supplemental Table 2). Results were similar for the presence of moderate or severe CAC on CT imaging (SHR 3.16, 95% CI 1.29 – 7.78, *p* = 0.012). Cumulative incidence of MACE stratified by both functional testing results and the presence of CAC on CT imaging are shown in Fig. 3.

**Table 3:** Test results and outcomes

	**Total Cohort *n* = 159**
**Abnormal functional testing**	17 (10.7%)
Positive	12 (7.5%)
Inconclusive	5 (3.1%)
**CAC present**	59 (37.1%)
Mild	42 (26.4%)
Moderate	15 (9.4%)
Severe	2 (1.3%)
**Rest left ventricular ejection fraction (%)**^a^	60 (55 – 65)
**Stress left ventricular ejection fraction (%)**^b^	69.5 (61 – 74)
**2019 ACC/AHA Guideline on the Primary Prevention of CV Disease recommended discussion regarding statin therapy**	23 (15.2%)
**MACE during follow-up period**	38 (23.9%)
Coronary revascularization	18
Hospitalization for heart failure	9
Hospitalization for nonfatal myocardial infarction	5
Hospitalization for ischemic stroke	4
Cardiovascular death	2
**Years to MACE from functional testing**	3.3 (0.9 – 6.9)
**Years to MACE from HL diagnosis**	30.5 (21 – 35)

Values are presented as median (interquartile range) or n (%) as appropriate

*CAC* Coronary artery calcifications, *HL* Hodgkin lymphoma, *MACE* Major adverse cardiovascular event

^a^Rest left ventricular ejection fraction available for 71 patients

^b^Stress left ventricular ejection fraction available for 110 patients

### Guideline-based risk assessment for CAD

Among the 159 patients in the study cohort, the 2019 ACC/AHA Guideline on the Primary Prevention of Cardiovascular Disease recommended a discussion regarding statin therapy in 23 (15.2%) patients (Table 3). Of the 128 patients for whom the primary prevention guidelines did not recommend a discussion regarding statin therapy, 38 (29.7%) had CAC present on CT imaging (Supplemental Table 1).

Additionally, 16 patients in the study cohort (10.1%) were eligible for cardiovascular risk assessment by the CCSS Cardiovascular Risk Calculator. This risk calculator deemed two (12.5%) patients as moderate/high risk and the remaining 14 (87.5%) patients as high risk for developing ischemic heart disease. The 2019 ACC/AHA primary prevention guidelines did not recommend statin therapy for any of the 16 patients, and none had abnormal functional testing. Four (25.0%) had CAC present on CT imaging. Three of the 16 patients (18.8%) experienced a MACE during the follow-up period.

### Diagnostic test characteristics

**Fig. 4:**
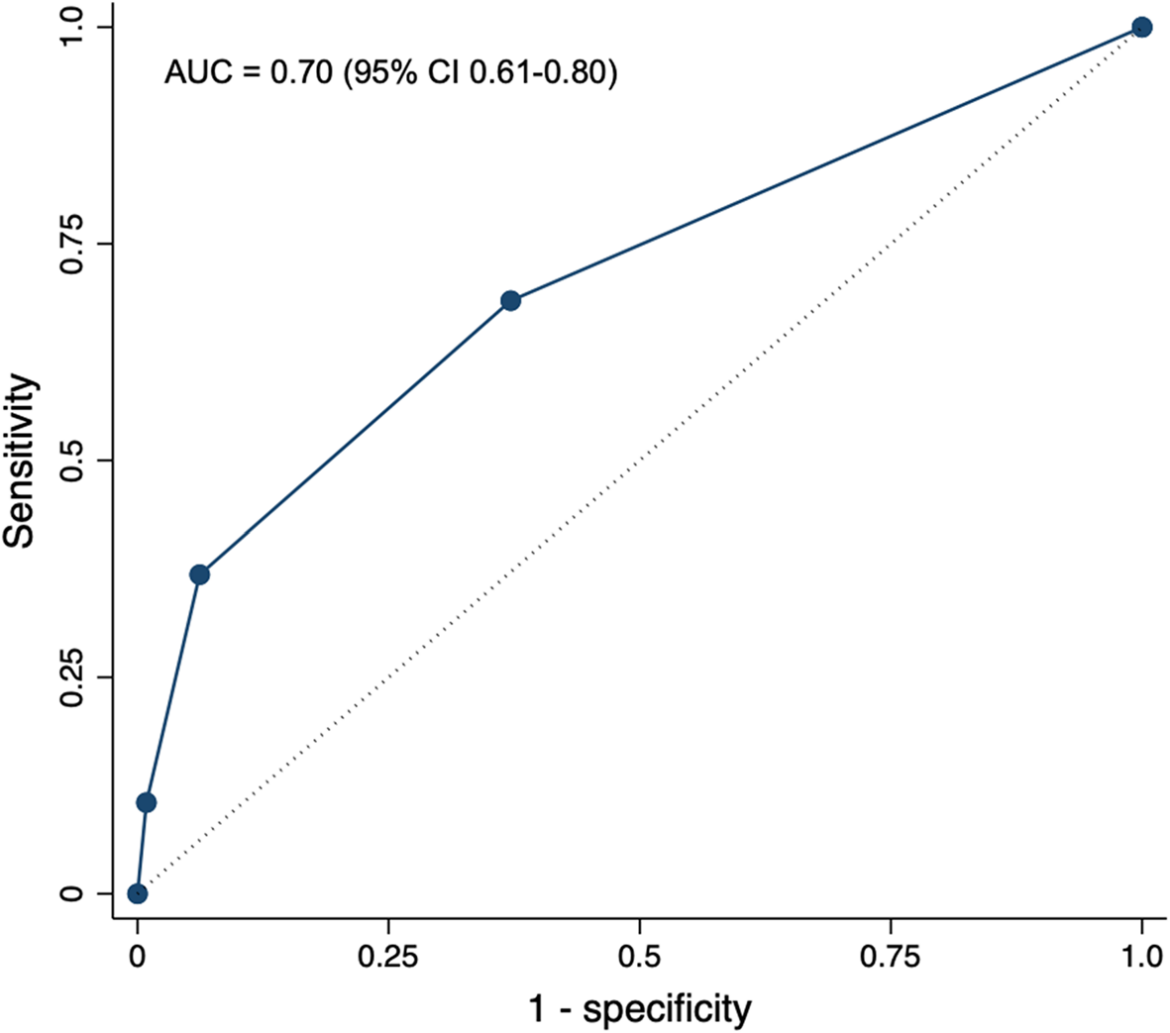
Receiver Operating Characteristic Curve: Entire Cohort. Receiver operating characteristic (ROC) curve with area under the curve (AUC) values for the combined coronary artery disease assessment strategy (functional testing, coronary artery calcification assessment, and guideline-recommend statin therapy discussion with the number of combined abnormal results (zero, one, two, or three) as the cut points) in the entire cohort. Only data from those patients who were assessable by all three strategies (*n* = 151) were included in the ROC analyses. CAC = coronary artery calcification. CI = confidence interval

Abnormal functional testing had the highest specificity (94.2% (95% CI 88.4%—97.6%)) and positive likelihood ratio (4.55 (95% CI 1.86 – 11.13)) while presence of CAC on CT imaging had the highest sensitivity (63.2% (95% CI 46.0%—78.2%)) and lowest negative likelihood ratio (0.52 (95% CI 0.34 – 0.80)) (Table 4). Like abnormal functional testing, primary prevention guideline recommendations had a high specificity (88.5% (95% CI 81.1%—93.7%)), but the negative likelihood ratio was not as low (0.83 (95% CI 0.68—1.02). ROC curve is shown in Fig. 4.

**Table 4:** Diagnostic Characteristics of Functional Testing, Semi-Quantitative Coronary Artery Calcification Assessment, and Primary Prevention Guidelines in the Entire Cohort

Full Cohort (*n* = 159)	MACE	No MACE	Sensitivity (95% CI)	Specificity (95% CI)	PPV (95% CI)	NPV (95% CI)	PLR (95% CI)	NLR (95% CI)
**Functional Study**			26.3% (13.4%—43.1%)	94.2% (88.4%—97.6%)	58.8% (36.9%—77.8%)	80.3% (77.0%—83.2%)	4.55 (1.86 – 11.13)	0.78 (0.64 – 0.95)
Abnormal	10	7						
Normal	28	114						
**Coronary Artery Calcifications (CAC)**			63.2% (46.0%—78.2%)	71.1% (62.1%—79.0%)	40.7% (32.1%—49.8%)	86.0% (80.0%—90.4%)	2.18 (1.51 – 3.16)	0.52 (0.34 – 0.80)
Present	24	35						
Absent	14	86						
**Moderate or Severe CAC**			29.0% (15.4%—45.9%)	95.0% (89.5%—98.2%)	64.7% (42.1%—82.2%)	81.0% (77.6%—84.0%)	5.84 (2.31 – 14.73)	0.75 (0.61 – 0.92)
Present	11	6						
Absent	27	115						
**2019 ACC/AHA Guideline**^a^			26.3% (13.4%—43.1%)	88.5% (81.1%—93.7%)	43.5% (26.9%—61.7%)	78.1% (74.5%—81.4%)	2.29 (1.09 – 4.78)	0.83 (0.68 – 1.02)
Recommend statin therapy discussion	10	13						
Do not recommend statin therapy discussion	28	100						

*ACC* American College of Cardiology, *AHA* American Heart Association, *CI* Confidence interval, *MACE* Major adverse cardiovascular event, *PPV* Positive predictive value, *NPV* Negative predictive value, *PLR* Positive likelihood ratio, *NLR* Negative likelihood ratio

^a^2019 ACC/AHA Guideline on the Primary Prevention of CV Disease

### Symptomatic and asymptomatic subgroups referred for functional testing

**Fig. 5:**
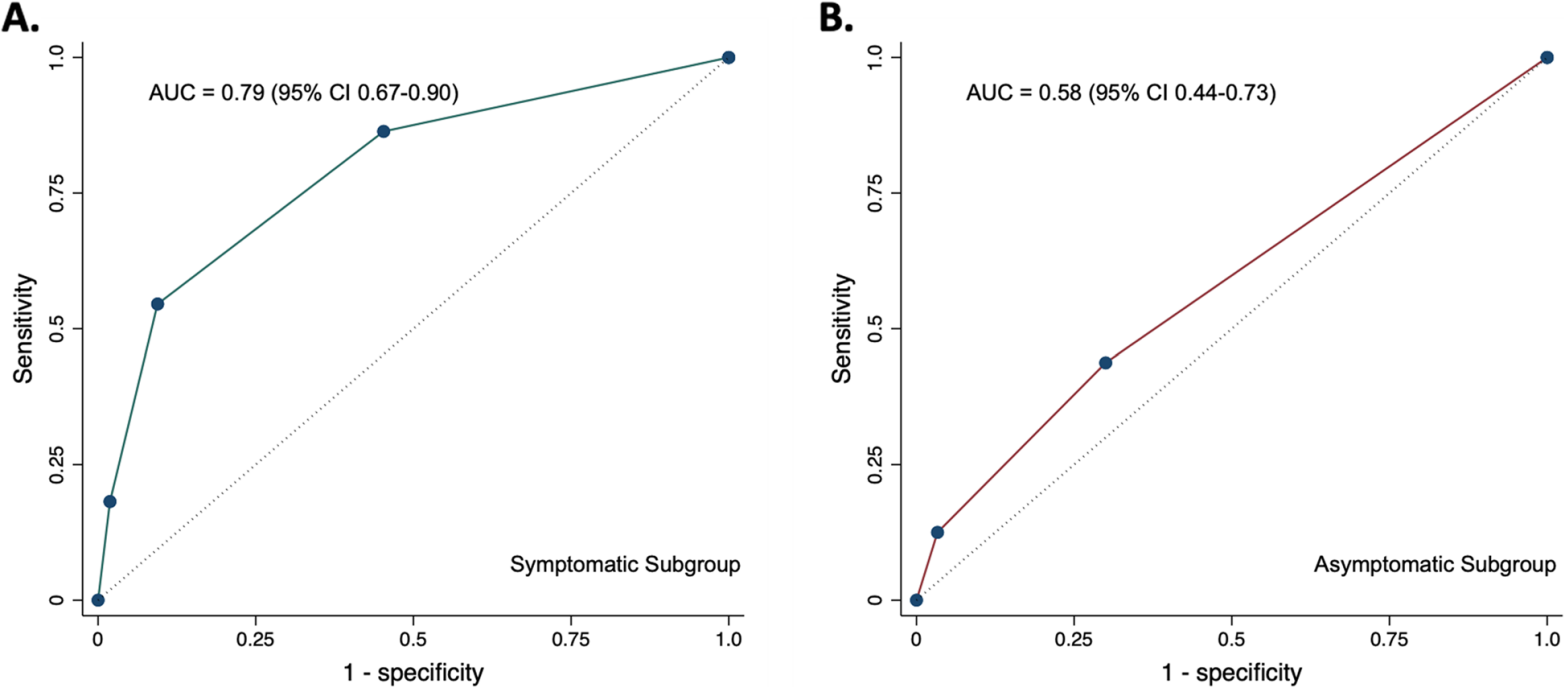
Receive Operating Characteristic Curves: Symptomatic and Asymptomatic Subgroups. Receiver operating characteristic (ROC) curves with area under the curve (AUC) values for the combined coronary artery disease assessment strategy (functional testing, coronary artery calcification assessment, and guideline-recommend statin therapy discussion with the number of combined abnormal results (zero, one, two, or three) as the cut points) in the A. subgroup of symptomatic patients referred for testing and B. subgroup of asymptomatic patients referred for coronary artery disease screening. Only data from those patients who were assessable by all three strategies (*n* = 74 for the symptomatic subgroup and *n* = 77 for the asymptomatic subgroup) were included in the ROC analyses. CAC = coronary artery calcification. CI = confidence interval

Among the 82 symptomatic patients referred for functional testing for evaluation of CAD, 14 (17.1%) had an abnormal functional study. Of the 68 who had normal functional testing, 29 (42.6%) had CAC present on CT imaging (Supplemental Table 3). Among the 82 patients in this subgroup, the 2019 ACC/AHA Guideline on the Primary Prevention of Cardiovascular Disease recommended a discussion regarding statin therapy in 18 (24.0%). Abnormal functional testing had the highest specificity (91.7% (95% CI 81.6%—97.2%)) in this subgroup (Table 5). Both abnormal functional testing and presence of CAC were associated with MACE in this subgroup (Supplemental Table 4). ROC curve for this subgroup is shown in Fig. 5A.

**Table 5:** Diagnostic Characteristics of Functional Testing, Semi-Quantitative Coronary Artery Calcification Assessment, and Primary Prevention Guidelines in the Subgroup of Patients Referred for Functional Testing For Symptoms

Symptomatic Cohort (*n* = 82)	MACE	No MACE	Sensitivity (95% CI)	Specificity (95% CI)	PPV (95% CI)	NPV (95% CI)	PLR (95% CI)	NLR (95% CI)
**Functional Study**			40.9% (20.7%—63.7%)	91.7% (81.6%—97.2%)	64.3% (40.4%—82.7%)	80.9% (74.8%—85.8%)	4.91 (1.85 – 13.05)	0.64 (0.45 – 0.92)
Abnormal	9	5						
Normal	13	55						
**Coronary Artery Calcifications (CAC)**			81.8% (59.7%—94.8%)	66.7% (53.3%—78.3%)	47.4% (37.4%—57.5%)	90.9% (80.2%—96.1%)	2.45 (1.63 – 3.69)	0.27 (0.11 – 0.67)
Present	18	20						
Absent	4	40						
**Moderate or Severe CAC**			45.5% (24.4%—67.8%)	95.0% (86.1%—99.0%)	76.9% (50.3%—91.7%)	82.6% (76.4%—87.5%)	9.09 (2.75 – 30.01)	0.57 (0.39 – 0.84)
Present	10	3						
Absent	12	57						
**2019 ACC/AHA Guideline**^a^			36.4% (17.2%—59.3%)	81.1% (68.0%—90.6%)	44.4% (26.7%—63.7%)	75.4% (68.6%—81.2%)	1.93 (0.88 – 4.23)	0.78 (0.56 – 1.10)
Recommend statin therapy discussion	8	10						
Do not recommend statin therapy discussion	14	43						

*ACC* American College of Cardiology, *AHA* American Heart Association, *CAD* Coronary artery disease, *CI* Confidence interval, *MACE* Major adverse cardiovascular event, *PPV* Positive predictive value, *NPV* Negative predictive value, *PLR* Positive likelihood ratio, *NLR* Negative likelihood ratio

^a^2019 ACC/AHA Guideline on the Primary Prevention of CV Disease

Among the 77 asymptomatic patients referred for functional testing for CAD screening, three (3.9%) had an abnormal functional study. Of the 74 who had normal functional testing, 21 (28.4%) had CAC present on CT imaging (Supplemental Table 5). Among the 77 patients in this subgroup, the 2019 ACC/AHA Guideline on the Primary Prevention of Cardiovascular Disease recommended a discussion regarding statin therapy in five (6.6%) patients. Of the 71 patients in this subgroup for whom the primary prevention guidelines did not recommend a discussion regarding statin therapy, 17 (23.9%) had CAC present on CT imaging (Supplemental Table 5). There was no significant association between abnormal functional testing or presence of CAC and MACE in this subgroup (Supplemental Table 6). Abnormal functional testing had the highest specificity (96.7% (95% CI 88.7%—99.6%)) in this subgroup (Table 6). However, the positive likelihood ratio was highest for the primary prevention guidelines (2.50 (95% CI 0.46 – 13.7)) with a similar specificity (95.0% (86.1%—99.0%)). The presence of CAC on CT imaging had the highest sensitivity (37.5% (95% CI 15.2%—64.6%)) and lowest negative likelihood ratio (0.83 (95% CI 0.55 – 1.24)) (Table 5). ROC curve for this subgroup is shown in Fig. 5B.

**Table 6:** Diagnostic Characteristics of Functional Testing, Semi-Quantitative Coronary Artery Calcification Assessment, and Primary Prevention Guidelines in the Subgroup of Patients Referred for Functional Testing For Asymptomatic Coronary Artery Disease Screening

Asymptomatic Cohort (*n* = 77)	MACE	No MACE	Sensitivity (95% CI)	Specificity (95% CI)	PPV (95% CI)	NPV (95% CI)	PLR (95% CI)	NLR (95% CI)
**Functional Study**			6.3% (0.2%—30.2%)	96.7% (88.7%—99.6%)	33.3% (4.6%—83.8%)	79.7% (77.5%—81.8%)	1.91 (0.18 – 19.72)	0.97 (0.85 – 1.11)
Abnormal	1	2						
Normal	15	59						
**Coronary Artery Calcifications (CAC)**			37.5% (15.2%—64.6%)	75.4% (62.7%—85.5%)	28.8% (15.6%—46.4%)	82.1% (75.4%—87.3%)	1.52 (0.71 – 3.29)	0.83 (0.55 – 1.24)
Present	6	15						
Absent	10	46						
**Moderate or Severe CAC**			6.3% (0.2%—30.2%)	95.1% (86.3%—99.0%)	25.0% (3.6%—75.0%)	79.5% (77.1%—81.6%)	1.27 (0.14 – 11.41)	0.99 (0.86 – 1.13)
Present	1	3						
Absent	15	58						
**2019 ACC/AHA Guideline**^a^			12.5% (1.6%—38.4%)	95.0% (86.1%—99.0%)	40.0% (10.8%—78.5%)	80.3% (77.0%—83.2%)	2.50 (0.46 – 13.7)	0.92 (0.76 – 1.12)
Recommend statin therapy discussion	2	3						
Do not recommend statin therapy discussion	14	57						

*ACC* American College of Cardiology, *AHA* American Heart Association, *CAD* Coronary artery disease, *CI* Confidence interval, *MACE* Major adverse cardiovascular event, *PPV* Positive predictive value, *NPV* Negative predictive value, *PLR* Positive likelihood ratio, *NLR* Negative likelihood ratio

^a^2019 ACC/AHA Guideline on the Primary Prevention of CV Disease

## Discussion

In this study that included HL survivors treated with chest radiation who were clinically referred for both functional imaging for evaluation of CAD and CT chest (for a range of reasons) within a 12-month period, we found that an abnormal functional study and primary prevention guideline-recommended discussion regarding statin therapy had high specificity, but low sensitivity for MACE during the follow-up period. The presence of CAC on CT imaging had higher sensitivity and a lower negative likelihood ratio, but lower specificity. CAC was present in 35.2% of patients with a normal functional study and 29.7% of patients for whom the primary prevention guidelines did not recommend discussion regarding statin therapy, identifying CAC on CT imaging as a potential imaging biomarker to utilize in primary prevention strategies in HL survivors.

Importantly, the data from the asymptomatic subgroup (*n* = 77) suggest that this specific population may benefit from a different strategy than that typically utilized for patients with symptoms that may be from CAD [18]. Notably, diagnostic characteristics of all three primary prevention strategies (functional testing, CAC by CT imaging, and primary prevention guideline-recommended statin therapy discussion) had poor sensitivities and negative likelihood ratios for this subgroup. As a comparison, a recent prospective comparative effectiveness study that studied 475 patients with stable chest pain and intermediate pre-test probability of obstructive CAD found that myocardial perfusion imaging had a sensitivity of 74% (and specificity of 73%) for significant CAD by invasive coronary angiography [19]. A recent multidisciplinary expert statement from the International Cardio-Oncology Society emphasized the importance of looking for incidental CAC on CT imaging to guide primary prevention statin therapy in cancer survivors treated with RT [20]. However, the results of the current study suggest that this strategy may miss patients who would potentially benefit from primary prevention therapy, suggesting that both calcified and non-calcified plaque play an important role in the pathophysiology of RT associated CAD. Additionally, the 2019 ACC/AHA Guideline on the Primary Prevention of Cardiovascular Disease had a low sensitivity for predicted MACE in this cohort, perhaps because this guideline does not include a history of chest RT as a risk-enhancing factor [6]. Although the CCSS Cardiovascular Risk Calculator [12] is sometimes extended for use in this population by clinicians, it is notable that only 10.1% of our study cohort fit the intended inclusion criteria for this tool. These findings highlight the need for the development of a cardiovascular risk calculator tool specific to this population and for the evaluation of other testing modalities to better identify those patients who might benefit from primary prevention strategies. Lastly, it is important to note there are limited data to demonstrate that statin therapy reduces incident MACE in patients at risk for radiation-associated CAD. Further studies are also needed to evaluate the benefit of potential primary prevention treatment strategies in this patient population.

Based on the data from the current study, we suggest the following algorithms for CAD evaluation in survivors of HL treated with chest radiation therapy:

### Symptomatic: diagnostic algorithm

For symptomatic patients, referral for functional testing or coronary computed tomography angiography (CCTA) can be made based on patient and provider preferences and according to contemporary consensus guidelines [18]. Notably, the cardiovascular imaging community has put reducing radiation exposure during nuclear cardiology and cardiac CT at the forefront of patient-centered initiatives [21–23]. Patients with abnormal functional testing results or obstructive CAD by CCTA can be considered for invasive coronary angiography if appropriate. Statin therapy discussion should be initiated for patients with abnormal functional testing and obstructive or non-obstructive CAD via CCTA. For patients without calcified or non-calcified coronary atherosclerosis via CCTA, statin therapy can be discussed if the primary prevention guidelines recommend statin therapy. Otherwise, it can be deferred. For patients with normal functional testing results, statin therapy discussion should be initiated if the 2019 ACC/AHA primary prevention guidelines recommend statin therapy. If the guidelines do not, a CCTA can be considered if clinical suspicion for CAD remains, or prior imaging can be reviewed for incidental CAC by CT chest. If CAD is present on CCTA, or incidental CAC is present on chest CT, statin therapy discussion should be initiated.

### Asymptomatic: primary prevention algorithm

Given the poor sensitivity of functional testing and guideline-directed primary prevention statin discussions, and modest sensitivity of CAC in the current study for asymptomatic HL survivors who are due for guideline-recommended screening for CAD, we recommend that patients who meet criteria for statin therapy discussion by the 2019 ACC/AHA primary prevention guidelines be initiated on statin therapy. Patients who do not meet these criteria may be referred for CCTA (barring contraindications). Discussion regarding statin therapy should be considered if calcified or non-calcified coronary atherosclerosis is identified.

### Study limitations

The current study has important limitations. It is a single-center, observational study with modest sample size in which the population consisted of patients referred clinically for functional testing and chest CT. Since inclusion criteria included functional testing and a chest CT within 12 months, the cohort inherently includes patients with secondary thoracic malignancies and/or cardiopulmonary symptoms. CAC was assessed semi-quantitatively and not via formal calcium scoring in most cases, and therefore we could not use risk calculators that incorporate Agatston scores [24]. In addition, because CAC was assessed visually on non-gated CT scans, it is possible that patients with a mild amount of calcified plaque were categorized as normal. As such, the sensitivity of formal CAC assessment in this population may be higher than reported in our study. However, the semi-quantitative approach is supported by societal guidelines [25] and we followed previously published methods [10, 11]. Twelve patients referred for perioperative testing were put in the symptomatic cohort as we assumed that a functional limitation or undocumented symptom was present to refer for testing. We used MACE as the gold standard for diagnostic evaluation since we did not have a diagnostic gold standard for flow-limiting, epicardial CAD such as invasive coronary angiography or CCTA for all patients. Finally, since endpoints were adjudicated using our local clinical and research records, it is possible that events outside of our healthcare system were not captured.

## Conclusions

In HL survivors treated with radiation therapy, both abnormal functional testing and primary prevention guideline-recommended statin therapy had high specificity for subsequent MACE, but presence of CAC on CT imaging had higher sensitivity. In a subgroup of HL survivors referred for asymptomatic CAD screening, the presence of CAC had only modest sensitivity for subsequent MACE. More work is needed regarding patient-centered screening and primary prevention strategies in cancer survivors treated with radiation therapy to the chest, including HL survivors.

## Supplementary Information

**Additional file 1.**

## Data Availability

The datasets used and/or analyzed during the current study are available from the corresponding author on reasonable request.

## Abbreviations

CAC: Coronary artery calcifications
CAD: Coronary artery disease
CT: Computed tomography
HL: Hodgkin lymphoma
LVEF: Left ventricular ejection fraction
MACE: Major adverse cardiovascular event
