# Supplementary material for: Functional testing, coronary artery calcifications, and outcomes in Hodgkin lymphoma survivors treated with chest radiation
Source: Cardiooncology. 2023 Jan 20;9:5. doi: 10.1186/s40959-023-00157-2 (PMC9854101; doi:10.1186/s40959-023-00157-2)

**Functional Testing, Coronary Artery Calcifications, and Outcomes in Hodgkin Lymphoma Survivors Treated with Chest Radiation**

Sanjay Divakaran, MD,^*^ Diana M. Lopez, MD,^*^ Sean M. Parks, BS, Jon Hainer, BS, Andrea K. Ng, MD, MPH, Ron Blankstein, MD, Marcelo F. Di Carli, MD, Anju Nohria, MD, MSc

**Supplemental Information**

**Table of Contents**

Supplemental Tables – 2

Supplemental Figure – 9

**Supplemental Table 1. Functional Study Results and Primary Prevention Guideline Recommendations Stratified by the Presence of Coronary Artery Calcifications in the Entire Cohort.**

| **Full Cohort (n=159)** | **CAC Absent**  **(n=100)** | **CAC Present**  **(n=59)** |
| --- | --- | --- |
| **Functional Study** |  |  |
| Normal (n=142) | 92 | 50 |
| Abnormal (n=17) | 8 | 9 |
| **2019 ACC/AHA Guideline on the Primary Prevention of Cardiovascular Disease** |  |  |
| Do not recommend statin therapy discussion (n=128) | 90 | 38 |
| Recommend statin therapy discussion (n=23) | 7 | 16 |

ACC = American College of Cardiology, AHA = American Heart Association, CAC = coronary artery calcifications.

**Supplemental Table 2. Competing Risk Regression to Study the Association Between Abnormal Functional Testing, Coronary Artery Calcification, and Incident Major Adverse Cardiovascular Events.** To study the baseline effect of abnormal functional testing and coronary artery calcification on major adverse cardiovascular events accounting for competing risk of death, Fine and Gray competing risks regression modeling was performed using the potential covariates listed below. Following this, multivariable adjustment was performed using Morise score, and covariates significantly associated with incident cardiovascular events not incorporated in the Morise score (excluding the 2019 ACC/AHA Guideline recommendations given overlap between the Morise score and the 10-year atherosclerotic cardiovascular disease (ASCVD) risk calculator). BMI = body mass index. CAD = coronary artery disease. CI = confidence interval. HL = Hodgkin lymphoma. LVEF = left ventricular ejection fraction. SD = standard deviation. SHR = subdistribution hazard ratio.

|  | **Univariable** | | | **Multivariable*** | | |
| --- | --- | --- | --- | --- | --- | --- |
| **Covariate** | **SHR** | **95% CI** | **p-value** | **SHR** | **95% CI** | **p-value** |
| **Abnormal functional testing** | 5.36 | 2.51 – 11.43 | <0.001 | 5.10 | 2.41 – 10.78 | <0.001 |
| **Coronary artery calcification present** | 3.75 | 1.95 – 7.20 | <0.001 | 3.58 | 1.35 – 9.47 | 0.010 |
| **2019 ACC/AHA Guideline on the Primary Prevention of Cardiovascular Disease recommends statin therapy discussion** | 2.74 | 1.35 – 5.54 | 0.005 |  |  |  |
| **Rest LVEF** | 1.07 | 0.96 – 1.19 | 0.209 |  |  |  |
| **Stress LVEF** | 0.97 | 0.94 – 1.00 | 0.081 |  |  |  |
| **Age at functional testing** | 1.03 | 1.00 – 1.07 | 0.036 |  |  |  |
| **Age at HL diagnosis** | 1.00 | 0.97 – 1.03 | 0.825 |  |  |  |
| **Years between HL diagnosis and functional testing** | 1.03 | 1.01 – 1.06 | 0.009 | 0.99 | 0.94 – 1.05 | 0.819 |
| **Treatment with anthracyclines** | 0.74 | 0.38 – 1.43 | 0.363 |  |  |  |
| **Treatment with mantle radiation or cumulative dose ≥35 Gy** | 1.01 | 0.32 – 3.19 | 0.987 |  |  |  |
| **Decade of radiation therapy** | 0.72 | 0.56 – 0.94 | 0.016 | 0.90 | 0.52 – 1.56 | 0.700 |
| **Female** | 0.83 | 0.43 – 1.58 | 0.564 |  |  |  |
| **Hypertension** | 1.98 | 1.04 – 3.77 | 0.036 |  |  |  |
| **Dyslipidemia** | 0.92 | 0.47 – 1.80 | 0.800 |  |  |  |
| **Diabetes** | 1.32 | 0.36 – 4.93 | 0.676 |  |  |  |
| **Former or current tobacco use** | 1.02 | 0.46 – 2.30 | 0.953 |  |  |  |
| **Family history of premature CAD** | 2.03 | 1.06 – 3.88 | 0.033 |  |  |  |
| **BMI ≥ 30 kg/m^2^** | 0.58 | 0.24 – 1.41 | 0.228 |  |  |  |
| **Morise score** | 1.10 | 0.98 – 1.23 | 0.092 | 0.99 | 0.86 – 1.14 | 0.866 |

*Multivariable analysis adjusted for abnormal functional testing, the presence of coronary artery calcification, years between Hodgkin lymphoma diagnosis and functional testing, decade of radiation therapy, and Morise score.

**Supplemental Table 3. Functional Study Results and Primary Prevention Guideline Recommendations Stratified by the Presence of Coronary Artery Calcifications in the Subgroup of Patients Referred for Functional Testing for Symptoms.**

| **Test Indication: Asymptomatic CAD Screening (n=82)** | **CAC Absent**  **(n=44)** | **CAC Present**  **(n=38)** |
| --- | --- | --- |
| **Functional Study** |  |  |
| Normal (n=68) | 39 | 29 |
| Abnormal (n=14) | 5 | 9 |
| **2019 ACC/AHA Guideline on the Primary Prevention of Cardiovascular Disease** |  |  |
| Do not recommend statin therapy discussion (n=57) | 36 | 21 |
| Recommend statin therapy discussion (n=18) | 6 | 12 |

ACC = American College of Cardiology, AHA = American Heart Association, CAC = coronary artery calcifications, CAD = coronary artery disease.

**Supplemental Table 4. Competing Risk Regression to Study the Association Between Abnormal Functional Testing, Coronary Artery Calcification, and Incident Major Adverse Cardiovascular Events in the Subgroup of Patients Referred for Functional Testing For Symptoms.** To study the baseline effect of abnormal functional testing and coronary artery calcification on major adverse cardiovascular events accounting for competing risk of death, Fine and Gray competing risks regression modeling was performed using the potential covariates listed below. Following this, multivariable adjustment was performed using Morise score, and covariates significantly associated with incident cardiovascular events not incorporated in the Morise score. BMI = body mass index. CAD = coronary artery disease. CI = confidence interval. HL = Hodgkin lymphoma. LVEF = left ventricular ejection fraction. SD = standard deviation. SHR = subdistribution hazard ratio.

|  | **Univariable** | | | **Multivariable*** | | |
| --- | --- | --- | --- | --- | --- | --- |
| **Covariate** | **SHR** | **95% CI** | **p-value** | **SHR** | **95% CI** | **p-value** |
| **Abnormal functional testing** | 5.44 | 2.35 – 12.62 | <0.001 | 5.03 | 1.99 – 12.74 | 0.001 |
| **Coronary artery calcification present** | 6.67 | 2.23 – 20.00 | 0.001 | 8.86 | 1.20 – 65.28 | 0.032 |
| **2019 ACC/AHA Guideline on the Primary Prevention of Cardiovascular Disease recommends statin therapy discussion** | 2.29 | 0.97 – 5.38 | 0.058 |  |  |  |
| **Rest LVEF** | 1.12 | 0.96 – 1.29 | 0.140 |  |  |  |
| **Stress LVEF** | 0.97 | 0.94 – 1.01 | 0.153 |  |  |  |
| **Age at functional testing** | 1.04 | 1.00 – 1.08 | 0.086 |  |  |  |
| **Age at HL diagnosis** | 0.99 | 0.95 – 1.03 | 0.588 |  |  |  |
| **Years between HL diagnosis and functional testing** | 1.04 | 1.01 – 1.07 | 0.016 | 0.99 | 0.93 – 1.07 | 0.880 |
| **Treatment with anthracyclines** | 0.47 | 0.19 – 1.12 | 0.090 |  |  |  |
| **Treatment with mantle radiation or cumulative dose ≥35 Gy** | 2.43 | 0.32 – 18.42 | 0.390 |  |  |  |
| **Decade of radiation therapy** | 0.71 | 0.52 – 0.98 | 0.036 | 1.08 | 0.54 – 2.18 | 0.829 |
| **Female** | 0.91 | 0.38 – 2.20 | 0.831 |  |  |  |
| **Hypertension** | 1.79 | 0.78 – 4.10 | 0.170 |  |  |  |
| **Dyslipidemia** | 0.81 | 0.34 – 1.90 | 0.623 |  |  |  |
| **Diabetes** | 0.00 |  |  |  |  |  |
| **Former or current tobacco use** | 0.70 | 0.15 – 3.23 | 0.646 |  |  |  |
| **Family history of premature CAD** | 2.23 | 0.85 – 5.82 | 0.102 |  |  |  |
| **BMI ≥ 30 kg/m^2^** | 0.38 | 0.09 – 1.60 | 0.188 |  |  |  |
| **Morise score** | 1.06 | 0.92 – 1.21 | 0.412 | 0.92 | 0.77 – 1.09 | 0.317 |

*Multivariable analysis adjusted for abnormal functional testing, the presence of coronary artery calcification, years between Hodgkin lymphoma diagnosis and functional testing, decade of radiation therapy, and Morise score.

**Supplemental Table 5. Functional Study Results and Primary Prevention Guideline Recommendations Stratified by the Presence of Coronary Artery Calcifications in the Subgroup of Patients Referred for Functional Testing For Asymptomatic Coronary Artery Disease Screening.**

| **Test Indication: Asymptomatic CAD Screening (n=77)** | **CAC Absent**  **(n=56)** | **CAC Present**  **(n=21)** |
| --- | --- | --- |
| **Functional Study** |  |  |
| Normal (n=74) | 53 | 21 |
| Abnormal (n=3) | 3 | 0 |
| **2019 ACC/AHA Guideline on the Primary Prevention of Cardiovascular Disease** |  |  |
| Do not recommend statin therapy discussion (n=71) | 54 | 17 |
| Recommend statin therapy discussion (n=5) | 1 | 4 |

ACC = American College of Cardiology, AHA = American Heart Association, CAC = coronary artery calcifications, CAD = coronary artery disease.

**Supplemental Table 6. Competing Risk Regression to Study the Association Between Abnormal Functional Testing, Coronary Artery Calcification, and Incident Major Adverse Cardiovascular Events in the Subgroup of Patients Referred for Functional Testing For Asymptomatic Coronary Artery Disease Screening.** To study the baseline effect of abnormal functional testing and coronary artery calcification on major adverse cardiovascular events accounting for competing risk of death, Fine and Gray competing risks regression modeling was performed using the potential covariates listed below. Multivariable adjustment was not performed given the lack of significant univariable association. BMI = body mass index. CAD = coronary artery disease. CI = confidence interval. HL = Hodgkin lymphoma. LVEF = left ventricular ejection fraction. SD = standard deviation. SHR = subdistribution hazard ratio.

|  | **Univariable** | | |
| --- | --- | --- | --- |
| **Covariate** | **SHR** | **95% CI** | **p-value** |
| **Abnormal functional testing** | 2.03 | 0.27 – 15.53 | 0.493 |
| **Coronary artery calcification present** | 1.85 | 0.69 – 4.94 | 0.222 |
| **2019 ACC/AHA Guideline on the Primary Prevention of Cardiovascular Disease recommends statin therapy discussion** | 2.28 | 0.64 – 8.12 | 0.204 |
| **Rest LVEF** | 1.02 | 0.89 – 1.17 | 0.738 |
| **Stress LVEF** | 0.97 | 0.91 – 1.04 | 0.439 |
| **Age at functional testing** | 1.02 | 0.97 – 1.08 | 0.404 |
| **Age at HL diagnosis** | 1.01 | 0.97 – 1.06 | 0.548 |
| **Years between HL diagnosis and functional testing** | 1.01 | 0.97 – 1.06 | 0.493 |
| **Treatment with anthracyclines** | 1.45 | 0.49 – 4.30 | 0.504 |
| **Treatment with mantle radiation or cumulative dose ≥35 Gy** | 0.43 | 0.11 – 1.64 | 0.215 |
| **Decade of radiation therapy** | 0.83 | 0.53 – 1.29 | 0.415 |
| **Female** | 0.69 | 0.26 – 1.82 | 0.453 |
| **Hypertension** | 1.71 | 0.57 – 5.14 | 0.339 |
| **Dyslipidemia** | 0.95 | 0.32 – 2.80 | 0.929 |
| **Diabetes** | 3.46 | 2.07 – 5.76 | <0.001 |
| **Former or current tobacco use** | 1.66 | 0.59 – 4.66 | 0.334 |
| **Family history of premature CAD** | 2.49 | 0.95 – 6.52 | 0.064 |
| **BMI ≥ 30 kg/m^2^** | 0.89 | 0.29 – 2.78 | 0.844 |
| **Morise score** | 1.16 | 0.93 – 1.43 | 0.183 |

**Supplemental Figure 1. Study Cohort Derivation.** CAD = coronary artery disease. CT = computed tomography. HL = Hodgkin lymphoma. ICD = International Classification of Diseases. OHT = orthoptic heart transplantation. RT = radiation therapy.


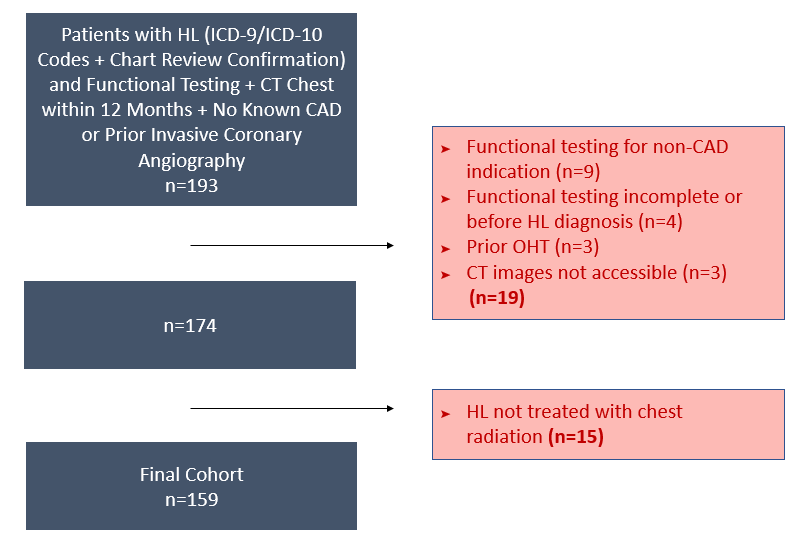

Supplement: Supplementary file 1 — Additional file 1. [file 40959_2023_157_MOESM1_ESM.docx]
